# Supplementary material for: Neutralization of Omicron sublineages and Deltacron SARS-CoV-2 by three doses of BNT162b2 vaccine or BA.1 infection
Source: Emerg Microbes Infect. 2022 Jul 27;11(1):1828–32. doi: 10.1080/22221751.2022.2099305 (PMC9331225; doi:10.1080/22221751.2022.2099305)
Supplement: Supplemental Material [file TEMI_A_2099305_SM2092.docx]

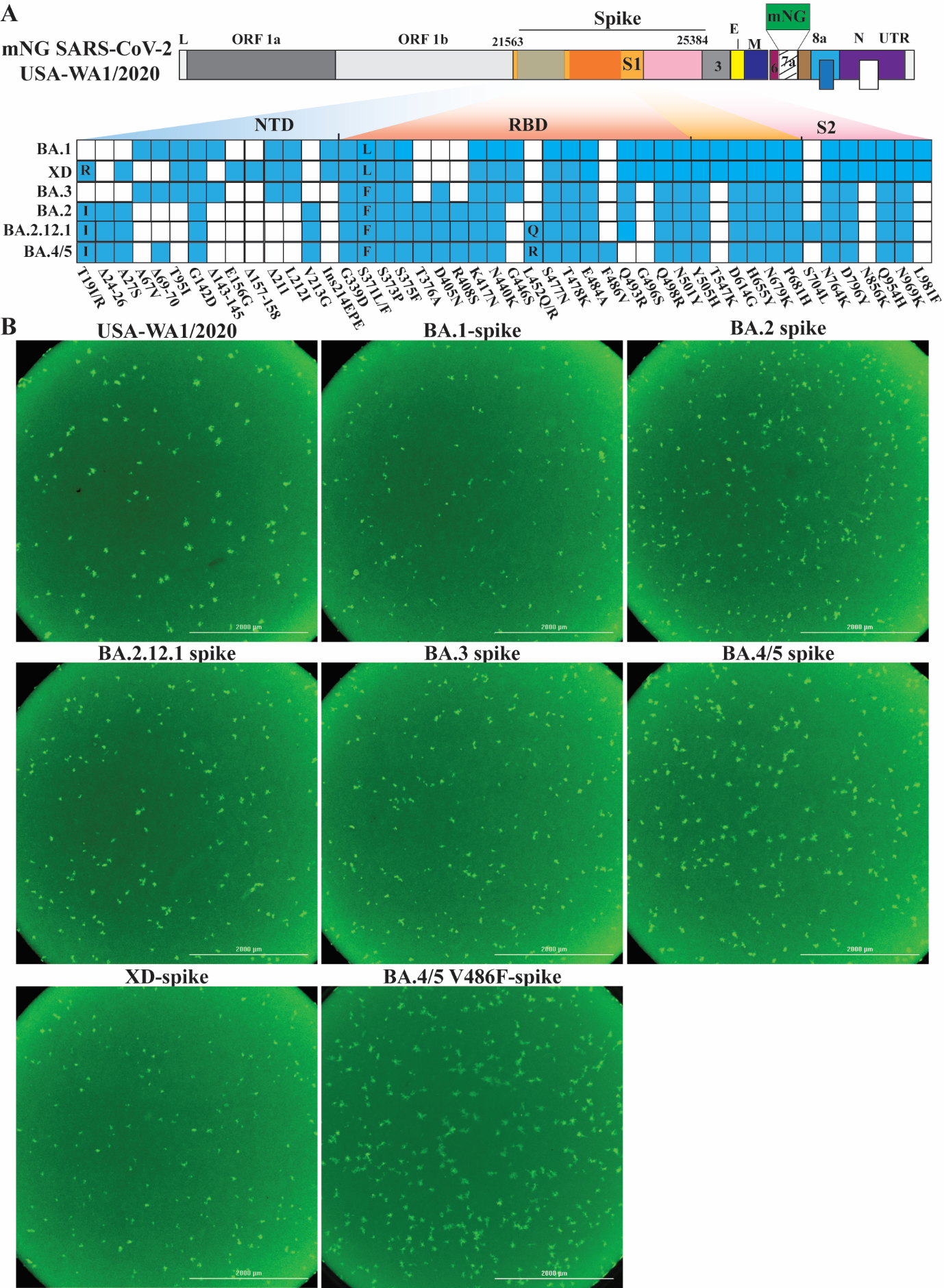


**Figure S1.** **Construction of Omicron sublineage BA.1-, BA.2-, BA.2.12.1-, BA.3-, BA.4/5-, and Deltacron XD-spike mNG SARS-CoV-2s.** (**A**) Omicron sublineage- and Deltacron XD-spike mNG SARS-CoV-2s. mNG USA-WA1/2020 SARS-CoV-2 was used to engineer Omicron sublineage- and Deltacron XD-spike SARS-CoV-2s. The mNG reporter gene was engineered at the open-reading-frame-7 (ORF7) of the USA-WA1/2020 genome.^1^ Amino acid mutations, deletions (∆), and insertions (Ins) are indicated for variant spikes in reference to the USA-WA1/2020 spike; the wild-type and mutant amino acids are indicated in white and blue, respectively. L: leader sequence; ORF: open reading frame; NTD: N-terminal domain of S1; RBD: receptor binding domain of S1; S: spike glycoprotein; S1: N-terminal furin cleavage fragment of S; S2: C-terminal furin cleavage fragment of S; E: envelope protein; M: membrane protein; N: nucleoprotein; UTR: untranslated region. (**B**) Fluorescent foci formed by USA-WA1/2020, BA.1-, BA.2-, BA.2.12.1-, BA.3-, BA.4/5-, XD-, and BA.4/5 V486F-spike mNG SARS-CoV-2s. The images were taken at 16 h after Vero E6 cells were infected with the indicated viruses in 96-well plates. Representative images are presented for each virus.


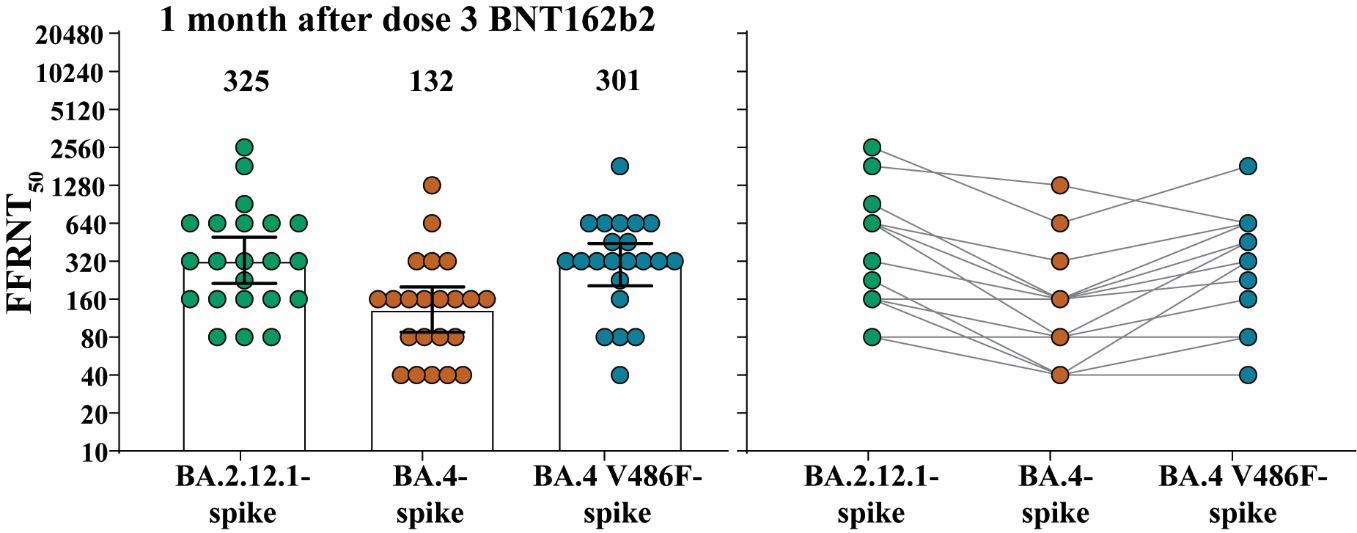


**Figure S2.** Neutralization by sera collected at 1 month post dose 3 BNT162b2 vaccine. Scatterplot plot (left) and connected line plot (right) of neutralization titers against Omicron sublineage BA.2.12.1-, BA.4/5-, and BA.4/5 V486F-spike mNG SARS-CoV-2s. The GMTs for individual variant-spike mNG SARS-CoV-2s were determined by FFRNT. The bar heights and the numbers above indicate GMTs. The whiskers indicate 95% confidence intervals. The dotted lines indicate the limit of detection of FFRNT_50_. Statistical analysis was performed with the use of the Wilcoxon matched-pairs signed-rank test. The P values of the GMT differences between BA.2.12.1-spike and BA.4-spike, BA.4-spike and BA.4 V486F-spike, BA.2.12.1-spike and BA.4 V486F-spike are < 0.0001, 0.0009, 0.2578, respectively.

**Table S1. FFRNT_50_ values of BNT162b2-vaccinated sera**

| Subject ID | Age (Years) | Sex (F/M) | *FFRNT_50_ | | | | | | | | | | | | | | | | | | | | | |
| --- | --- | --- | --- | --- | --- | --- | --- | --- | --- | --- | --- | --- | --- | --- | --- | --- | --- | --- | --- | --- | --- | --- | --- | --- |
|  |  |  | USA-WA1/2022 | | | | BA.1-spike | | | BA.2-spike | | | BA.2.12.1-spike | | | BA.3-spike | | | BA.4/5-spike | | | XD-spike | | |
|  |  |  | ^&^Exp1 | ^&^Exp2 | Exp3 | GMT | ^&^Exp1 | Exp2 | GMT | ^&^Exp1 | Exp2 | GMT | Exp1 | Exp2 | GMT | ^&^Exp1 | Exp2 | GMT | Exp1 | Exp2 | GMT | Exp1 | Exp2 | GMT |
| 1 | 26 | F | 640 | 905 | 1280 | 905 | 320 | 113 | 453 | 320 | 320 | 320 | 320 | 320 | 320 | 226 | 226 | 226 | 160 | 160 | 160 | 320 | 453 | 381 |
| 2 | 28 | M | 1280 | 1810 | 2560 | 1810 | 320 | 640 | 320 | 320 | 320 | 320 | 320 | 320 | 320 | 113 | 160 | 135 | 160 | 160 | 160 | 160 | 320 | 226 |
| 3 | 35 | F | 1280 | 1280 | 1280 | 1280 | 640 | 40 | 761 | 453 | 57 | 381 | 320 | 453 | 381 | 320 | 320 | 320 | 113 | 80 | 95 | 320 | 640 | 453 |
| 4 | 35 | F | 1280 | 1280 | 1280 | 1280 | 320 | 640 | 453 | 226 | 320 | 269 | 226 | 320 | 269 | 226 | 320 | 269 | 20 | 28 | 24 | 320 | 320 | 320 |
| 5 | 38 | F | 1280 | 1810 | 2560 | 1810 | 640 | 1280 | 761 | 640 | 905 | 640 | 640 | 640 | 640 | 320 | 453 | 381 | 226 | 226 | 226 | 640 | 640 | 640 |
| 6 | 38 | F | 320 | 320 | 640 | 403 | 160 | 226 | 160 | 160 | 160 | 160 | 80 | 113 | 95 | 80 | 160 | 113 | 28 | 28 | 28 | 160 | 160 | 160 |
| 7 | 44 | F | 640 | 453 | 640 | 570 | 113 | 320 | 113 | 113 | 113 | 95 | 80 | 80 | 80 | 80 | 80 | 80 | 40 | 40 | 40 | 80 | 80 | 80 |
| 8 | 44 | F | 2560 | 1280 | 2560 | 2032 | 640 | 640 | 761 | 640 | 320 | 640 | 640 | 905 | 761 | 320 | 640 | 453 | 226 | 226 | 226 | 640 | 640 | 640 |
| 9 | 52 | F | 2560 | 1280 | 2560 | 2032 | 320 | 905 | 453 | 320 | 640 | 320 | 453 | 453 | 453 | 320 | 320 | 320 | 160 | 160 | 160 | 453 | 320 | 381 |
| 10 | 54 | M | 1280 | 1280 | 1280 | 1280 | 453 | 160 | 453 | 320 | 160 | 320 | 320 | 320 | 320 | 226 | 320 | 269 | 80 | 160 | 113 | 320 | 320 | 320 |
| 11 | 65 | M | 1280 | 640 | 1280 | 1016 | 226 | 453 | 226 | 226 | 160 | 190 | 160 | 160 | 160 | 113 | 113 | 113 | 80 | 80 | 80 | 160 | 160 | 160 |
| 12 | 65 | M | 5120 | 2560 | 2560 | 3225 | 640 | 905 | 905 | 1280 | 320 | 1280 | 1810 | 1810 | 1810 | 320 | 640 | 453 | 640 | 640 | 640 | 640 | 640 | 640 |
| 13 | 66 | F | 640 | 640 | 1280 | 806 | 160 | 1280 | 226 | 160 | 640 | 160 | 226 | 160 | 190 | 80 | 160 | 113 | 80 | 80 | 80 | 160 | 160 | 160 |
| 14 | 67 | M | 640 | 905 | 1280 | 905 | 320 | 320 | 381 | 160 | 320 | 160 | 160 | 160 | 160 | 113 | 160 | 135 | 28 | 20 | 24 | 160 | 226 | 190 |
| 15 | 68 | M | 640 | 905 | 1280 | 905 | 160 | 2560 | 160 | 113 | 1280 | 135 | 160 | 160 | 160 | 113 | 113 | 113 | 40 | 57 | 48 | 160 | 160 | 160 |
| 16 | 68 | F | 5120 | 2560 | 3620 | 3620 | 905 | 160 | 1076 | 640 | 160 | 640 | 640 | 905 | 761 | 640 | 640 | 640 | 160 | 160 | 160 | 640 | 905 | 761 |
| 17 | 68 | F | 640 | 640 | 640 | 640 | 160 | 320 | 226 | 113 | 160 | 113 | 160 | 160 | 160 | 80 | 80 | 80 | 40 | 40 | 40 | 160 | 160 | 160 |
| 18 | 69 | F | 1280 | 905 | 1280 | 1140 | 320 | 1280 | 320 | 226 | 1280 | 269 | 320 | 320 | 320 | 160 | 226 | 190 | 160 | 160 | 160 | 320 | 320 | 320 |
| 19 | 69 | F | 2560 | 1810 | 2560 | 2281 | 905 | 320 | 1076 | 640 | 320 | 761 | 640 | 640 | 640 | 640 | 640 | 640 | 160 | 160 | 160 | 640 | 640 | 640 |
| 20 | 70 | M | 10240 | 10240 | 7241 | 9123 | 1280 | 640 | 1810 | 1810 | 320 | 1522 | 2560 | 1810 | 2153 | 1810 | 1280 | 1522 | 640 | 640 | 640 | 1810 | 1810 | 1810 |
| 21 | 73 | M | 320 | 453 | 640 | 508 | 40 | 453 | 40 | 80 | 320 | 67 | 57 | 80 | 67 | 28 | 40 | 34 | 40 | 40 | 40 | 40 | 57 | 48 |
| 22 | 74 | F | 1280 | 1280 | 2560 | 1613 | 640 | 905 | 640 | 320 | 640 | 320 | 320 | 320 | 320 | 160 | 320 | 226 | 160 | 160 | 160 | 320 | 453 | 381 |
| ^†^GMT | -- | -- | 1280 | 1146 | 1596 | 1335 | 336 | 460 | 393 | 300 | 315 | 298 | 305 | 325 | 315 | 190 | 245 | 216 | 101 | 105 | 103 | 282 | 320 | 301 |
| ^^^95% CI | -- | -- | 866-1893 | 827-1589 | 1208-2109 | 972-1834 | 234-482 | 301-702 | 266-578 | 210-431 | 223-446 | 208-428 | 202-462 | 219-483 | 211-471 | 127-284 | 169-356 | 148-316 | 66-155 | 69-159 | 68-156 | 195-409 | 223-461 | 209-432 |

*Individual FFRNT_50_ value is the geometric mean of duplicate FFRNT results.

^#^The sera were collected at 1 month post dose 3 BNT162b2 vaccine, as recently reported.^2^

^†^Geometric mean neutralizing titer (GMT).

^^^95% confidence interval (95% CI) for the GMT.

^&^These data were reported previously.^3^

**Table S2. FFRNT_50_ values of Omicron sublineage BA.1-infected sera**

| **Serum ID** | **Age (years)** | **Gender (F/M)** | ***FFRNT_50_** | | | | | | | | | | | | | | | | | | | | | **Serum collection time (days post positive RT-PCR test)** |
| --- | --- | --- | --- | --- | --- | --- | --- | --- | --- | --- | --- | --- | --- | --- | --- | --- | --- | --- | --- | --- | --- | --- | --- | --- |
|  |  |  | **USA-WA1/2020** | | | **BA.1-spike** | | | **BA.2-spike** | | | **BA.2.12.1-spike** | | | **BA.3-spike** | | | **BA.4/5-spike** | | | **XD-spike** | | |  |
|  |  |  | ^&^Exp1 | Exp2 | GMT | ^&^Exp1 | Exp2 | GMT | ^&^Exp1 | Exp2 | GMT | Exp1 | Exp2 | GMT | ^&^Exp1 | Exp2 | GMT | Exp1 | Exp2 | GMT | Exp1 | Exp2 | GMT |  |
| 1 | 21-30 | M | ^10 | 10 | 10 | 80 | 113 | 95 | 160 | 40 | 80 | 40 | 28 | 34 | 20 | 28 | 24 | 10 | 10 | 10 | 80 | 113 | 95 | 26 |
| 2 | 41-50 | F | 10 | 10 | 10 | 80 | 113 | 95 | 57 | 57 | 80 | 40 | 20 | 28 | 40 | 40 | 40 | 10 | 10 | 10 | 80 | 80 | 80 | 33 |
| 3 | 81-90 | M | 10 | 10 | 10 | 113 | 160 | 135 | 80 | 40 | 34 | 57 | 40 | 48 | 28 | 20 | 24 | 10 | 10 | 10 | 80 | 113 | 95 | 21 |
| 4 | 11-20 | M | 14 | 10 | 12 | 113 | 80 | 95 | 160 | 40 | 40 | 40 | 40 | 40 | 14 | 14 | 14 | 10 | 10 | 10 | 40 | 57 | 48 | 16 |
| 5 | 31-40 | F | 10 | 10 | 10 | 160 | 320 | 226 | 113 | 80 | 57 | 80 | 80 | 80 | 40 | 57 | 48 | 20 | 20 | 20 | 160 | 160 | 160 | 16 |
| 6 | 21-30 | F | 10 | 10 | 10 | 160 | 160 | 160 | 28 | 40 | 28 | 40 | 28 | 34 | 20 | 40 | 28 | 10 | 10 | 10 | 80 | 80 | 80 | 28 |
| 7 | 1-10 | F | 10 | 10 | 10 | 160 | 160 | 160 | 10 | 113 | 80 | 80 | 80 | 80 | 57 | 80 | 67 | 40 | 40 | 40 | 160 | 160 | 160 | 43 |
| 8 | 61-70 | M | 10 | 10 | 10 | 160 | 80 | 113 | 226 | 40 | 57 | 40 | 40 | 40 | 20 | 28 | 24 | 10 | 10 | 10 | 80 | 80 | 80 | 40 |
| 9 | 81-90 | M | 20 | 10 | 14 | 226 | 160 | 190 | 1280 | 113 | 135 | 57 | 57 | 57 | 226 | 57 | 113 | 10 | 10 | 10 | 113 | 113 | 113 | 40 |
| 10 | 1-10 | F | 10 | 10 | 10 | 320 | 226 | 269 | 160 | 40 | 34 | 28 | 28 | 28 | 40 | 40 | 40 | 10 | 10 | 10 | 160 | 160 | 160 | 26 |
| 11 | 1-10 | F | 10 | 10 | 10 | 320 | 320 | 320 | 28 | 160 | 135 | 160 | 226 | 190 | 160 | 160 | 160 | 40 | 40 | 40 | 320 | 320 | 320 | 56 |
| 12 | 51-60 | F | 10 | 10 | 10 | 453 | 453 | 453 | 20 | 160 | 67 | 160 | 160 | 160 | 80 | 80 | 80 | 80 | 80 | 80 | 226 | 226 | 226 | 35 |
| 13 | 71-80 | M | 160 | 40 | 80 | 453 | 160 | 269 | 2560 | 10 | 10 | 10 | 10 | 10 | 20 | 14 | 17 | 10 | 10 | 10 | 57 | 40 | 48 | 17 |
| 14 | 21-30 | F | 20 | 10 | 14 | 640 | 453 | 538 | 40 | 113 | 226 | 113 | 160 | 135 | 160 | 160 | 160 | 14 | 10 | 12 | 226 | 320 | 269 | 62 |
| 15 | 51-60 | M | 10 | 10 | 10 | 905 | 905 | 905 | 40 | 160 | 80 | 160 | 80 | 113 | 113 | 113 | 113 | 10 | 10 | 10 | 640 | 640 | 640 | 29 |
| 16 | 71-80 | F | 10 | 10 | 10 | 1280 | 1810 | 1522 | 453 | 226 | 226 | 1280 | 2560 | 1810 | 320 | 453 | 381 | 226 | 226 | 226 | 2560 | 2560 | 2560 | 8 |
| 17 | 71-80 | F | 28 | 14 | 20 | 2560 | 1280 | 1810 | 453 | 320 | 381 | 320 | 453 | 381 | 640 | 453 | 538 | 57 | 40 | 48 | 1280 | 1280 | 1280 | 32 |
| 18 | 61-70 | M | 113 | 40 | 67 | 2560 | 1810 | 2153 | 640 | 640 | 905 | 320 | 453 | 381 | 1810 | 905 | 1280 | 80 | 113 | 95 | 1280 | 1280 | 1280 | 15 |
| 19 | 71-80 | F | 10 | 10 | 10 | 5120 | 7241 | 6089 | 113 | 905 | 761 | 640 | 640 | 640 | 1280 | 1810 | 1522 | 80 | 57 | 67 | 2560 | 5120 | 3620 | 16 |
| 20 | 81-90 | M | 40 | 113 | 67 | 14482 | 20480 | 17222 | 28 | 2560 | 2560 | 2560 | 3620 | 3044 | 2560 | 3620 | 3044 | 320 | 320 | 320 | 10240 | 14482 | 12177 | 13 |
| ^#^GMT | - | - | 16 | 13 | 15 | 445 | 415 | 430 | 115 | 113 | 110 | 109 | 109 | 109 | 102 | 102 | 102 | 25 | 24 | 25 | 269 | 299 | 284 | 25 |
| ^†^95% CI | - | - | 11-24 | 10-18 | 10-20 | 225-881 | 205-842 | 216-855 | 58-228 | 62-208 | 59-206 | 58-207 | 52-230 | 55-218 | 48-218 | 48-214 | 49-215 | 15-43 | 14-42 | 14-42 | 132-549 | 141-632 | 137-588 | 20-32 |

*Individual FFRNT_50_ value is the geometric mean of duplicate FFRNT results.

^FFRNT_50_ of <20 was treated as 10 for plot purpose and statistical analysis.

^#^Geometric mean neutralizing titer (GMT).

^†^95% confidence interval (95% CI) for the GMT.

^&^ These data were reported previously.^4^

**Table S3. FFRNT_50_ values of BNT162b2-vaccinated sera against BA.4/5 V486F-spike.**

| Subject ID | Age (Years) | Sex (F/M) | *FFRNT_50_ | | |
| --- | --- | --- | --- | --- | --- |
|  |  |  | BA.2.12.1-spike | BA.4/5-spike | BA.4/5 V486F-spike |
| 1 | 26 | F | 320 | 160 | 320 |
| 2 | 28 | M | 640 | 160 | 320 |
| 3 | 35 | F | 640 | 80 | 453 |
| 4 | 35 | F | 226 | 40 | 320 |
| 5 | 38 | F | 640 | 320 | 640 |
| 6 | 38 | F | 80 | 40 | 80 |
| 7 | 44 | F | 80 | 80 | 80 |
| 8 | 44 | F | 640 | 320 | 640 |
| 9 | 52 | F | 320 | 160 | 453 |
| 10 | 54 | M | 320 | 160 | 320 |
| 11 | 65 | M | 160 | 160 | 226 |
| 12 | 65 | M | 1810 | 1280 | 640 |
| 13 | 66 | F | 160 | 80 | 160 |
| 14 | 67 | M | 160 | 40 | 320 |
| 15 | 68 | M | 160 | 80 | 80 |
| 16 | 68 | F | 905 | 160 | 640 |
| 17 | 68 | F | 160 | 40 | 320 |
| 18 | 69 | F | 320 | 160 | 320 |
| 19 | 69 | F | 640 | 320 | 640 |
| 20 | 70 | M | 2560 | 640 | 1810 |
| 21 | 73 | M | 80 | 40 | 40 |
| 22 | 74 | F | 320 | 160 | 320 |
| ^†^GMT | -- | -- | 325 | 132 | 301 |
| ^^^95% CI | -- | -- | 213-496 | 87-201 | 203-444 |

*Individual FFRNT_50_ value is the geometric mean of duplicate FFRNT results.

^#^Geometric mean neutralizing titer (GMT).

^†^95% confidence interval (95% CI) for the GMT.

**References**

1 Xie, X. *et al.* An Infectious cDNA Clone of SARS-CoV-2. *Cell Host Microbe* **27**, 841-848 e843, doi:10.1016/j.chom.2020.04.004 (2020).

2 Falsey, A. R. *et al.* SARS-CoV-2 Neutralization with BNT162b2 Vaccine Dose 3. *N Engl J Med*, doi:10.1056/NEJMc2113468 (2021).

3 Kurhade, C. *et al.* Neutralization of Omicron BA.1, BA.2, and BA.3 SARS-CoV-2 by 3 doses of BNT162b2 vaccine. *Nature Communications*, In press (2022).

4 Zou, J. *et al.* Cross-neutralization of Omicron BA.1 against BA.2 and BA.3 SARS-CoV-2. *Nat Commun* **13**, 2956, doi:10.1038/s41467-022-30580-5 (2022).
